# Heterogeneous Comorbidity in Individuals With Different Phenotypes of Obesity

**DOI:** 10.7759/cureus.38995

**Published:** 2023-05-14

**Authors:** Albina R Nurieva, Swapnil D Parve, Albina V Sineglazova

**Affiliations:** 1 General Practice, Kazan State Medical University, Kazan, RUS; 2 Medicine, Datta Meghe Institute of Medical Sciences (Deemed to be University), Wardha, IND

**Keywords:** comorbidity, obesity phenotypes, visceral obesity, abdominal obesity, obesity

## Abstract

Introduction

The prevalence of obesity is steadily increasing worldwide. Obesity is one of the most potent risk factors for various diseases and is simultaneously a heterogeneous condition. Different types of obesity could be identified according to body mass index (BMI), waist circumference, and visceral fat level; these conditions may present individually or in combination and pose a risk of developing certain comorbidities. However, the current obesity classification systems do not allow for accurate diagnosis and prediction of the comorbidity risk of patients, which is crucial for their clinical management. This points to the importance of studying obesity phenotyping in the context of body composition. Our study aimed to determine the contribution of obesity phenotypes in forming various comorbidities.

Materials and methods

This case-control study was conducted at the Clinical and Diagnostic Center of the Aviastroitelny District, Kazan. Patients were selected based on BMI per inclusion and exclusion criteria. A total of 151 patients with a median age of 43 [34.5-50] years were included in the study. The participants were distributed into six groups according to BMI and a combination of abdominal obesity (AO) and excess visceral fat.

Results

The participants were distributed in the following phenogroups: The first group - normal BMI without AO and excess visceral fat (n=47; 31.1%); the second group - overweight without AO and excess visceral fat (n=26; 17.2%); the third group - normal BMI with AO and without excess visceral fat (n=11; 7.3%); fourth group - overweight with AO and without excess visceral fat (n=34; 22.5%); fifth group - general obesity with AO and without excess visceral fat (n=20; 13.2%); sixth group - general obesity with AO and excess visceral fat (n=13; 8.6%). The five most frequently observed conditions in the general cohort were dyslipidemia (71.5%; n=108), disorders of the gastrointestinal tract (53.0%; n=80), cardiovascular disease (46.4%; n=70), musculoskeletal diseases (40.4%; n=61) and impaired carbohydrate metabolism (25.2%; n=38). The median number of pathological combinations in the general cohort was 5 [IQR: 3-7]. As the group number increased, the median number of comorbidities also increased. While BMI was significantly associated only with arterial hypertension, the level of visceral fat was associated with most comorbidities (obstructive sleep apnea syndrome, non-alcoholic fatty liver disease, chronic pancreatitis, hypertriglyceridemia, and prediabetes), followed by abdominal obesity (gastroesophageal reflux disease, hypertriglyceridemia, arterial hypertension, hypercholesterolemia).

Conclusions

In working-age people, group 1 and 4 phenotypes were more frequent than others. Abdominal obesity and visceral fat were associated with the most comorbid conditions. However, the individual types of these comorbidities were not the same.

## Introduction

The prevalence of obesity is steadily increasing worldwide [1]. Obesity is one of the most potent risk factors for various diseases and is simultaneously a heterogeneous condition [2,3]. Different types of obesity can be distinguished based on body mass index (BMI), waist circumference, and visceral fat level; these conditions may present individually or in combination [4,5]. A variety of comorbidities are associated with the different forms of obesity. Even being overweight is a significant risk factor for cardiometabolic multimorbidity [6-8]. A large prospective study that lasted slightly over 12 years demonstrated that diseases such as diabetes, hypertension, sleep disorders, osteoarthritis, arrhythmias, bacterial infections, and asthma were most commonly associated with obesity and resulted in the development of multimorbidity [9]. Several studies have found that the presence of adipose cells in the liver, pancreas, pericardium, and epicardium increases the risk of developing certain comorbidities [10-12]. Liu et al. studied twelve obesity-related comorbidities and assessed the effects of various fat distributions (subcutaneous, visceral, android, and gynoid) on multimorbidity. They found that an increase in total abdominal fat did not appear to affect the risk of any comorbidity. This finding differs slightly from previous reports indicating that total abdominal fat is an independent risk factor for cardiovascular diseases. Visceral fat is primarily responsible for total abdominal fat formation, whereas subcutaneous fat has a protective effect [13]. Finally, the current obesity classification systems do not allow for the accurate diagnosis and prediction of the comorbidity risk of patients, which is crucial for their clinical management. This highlights the importance of studying obesity phenotyping in the context of body composition [14]. Our study aimed to determine the contribution of obesity phenotypes in the formation of various types of comorbidities.

## Materials and methods

Study design and population

This case-control study was conducted at the Clinical and Diagnostic Center (CDC) of the Aviastroitelny District, Kazan. Patients were selected based on their BMI (⅓ normal weight, ⅓ overweight, and ⅓ obese) according to the inclusion and exclusion criteria. The sample size was calculated using the application Epi Info v5.5.11 for iOS. A total of 151 patients (Females: 87; Males: 64) in the age group of 25 to 59 years, with a median age of 43 [34.5-50] years, were included in the study.

Inclusion criteria

Individuals aged 25-44 years and the presence of voluntary informed consent to participate in the study.

Exclusion criteria

Refusal of the subject to participate in the study; patients with mental illness hindering the interview; the presence of verified cardiometabolic diseases (type 2 diabetes mellitus, coronary artery disease, congestive heart failure, atrial fibrillation, peripheral artery disease, chronic kidney disease); antiphospholipid syndrome; autoimmune inflammatory diseases; the presence of verified oncology; decompensatory states of concomitant diseases or conditions (liver disease, kidney disease, etc.); acute infectious diseases; diseases of the endocrine system and other conditions that serve as a secondary cause of obesity; medical implants including a pacemaker, silicone implants, and metal prostheses; pregnant and lactating women.

Data collection

A detailed patient consultation was conducted with a thorough history and physical exam, including anthropometry. BMI was categorized according to the World Health Organization classification [15]. Abdominal obesity (AO) was defined either as a waist circumference (WC) ≥ 94 centimeters in men and ≥ 80 centimeters in women and/or a waist-to-hip ratio (WHR) greater than 0.9 in men or 0.85 in women [16]. Body composition was evaluated using the Tanita- BC-601 body composition monitor. A visceral fat rating of 1-12 was considered normal, whereas 13-59 was defined as excess visceral fat (EVF) level. The work-up included a complete blood count, lipid profile, fasting plasma glucose level, oral glucose tolerance test, and glycated hemoglobin (HbA1c) level. The participants were distributed into six groups according to BMI and combination of AO and EVF: first group - normal BMI without AO and EVF; second group - overweight without AO and EVF; third group - normal BMI with AO and without EVF; fourth group - overweight with AO and without EVF; fifth group - general obesity (GO) with AO and without EVF; and sixth group - GO with AO and EVF.

Assessment of comorbidity

The presence of disease was established based on the results of history, physical examination, and the analysis of medical records that reflected the International Classification of Diseases - 10 codes. Furthermore, we used the following definitions to establish the diagnoses. Patients with a previous diagnosis of arterial hypertension and/or those receiving antihypertensive medications were classified as hypertensive subjects. Patients with BP ranging from 130/85 mmHg to 139/89 mmHg were diagnosed with high normal BP. Furthermore, patients without any prior diagnosis of hypertension or hypertensive urgency and with BP ≥ 140/90 mmHg on physical examination were classified as suspected cases of hypertension and were invited for a follow-up to confirm the diagnosis of hypertension. Uncontrolled hypertension was defined as when target BP levels were not reached in patients receiving antihypertensive therapy [17]. Patients were considered to have dyslipidemia when they had one or more parameters suggestive of altered lipid profile, such as increased levels of total cholesterol (≥5.0 mmol/l), low-density lipoprotein cholesterol (≥3.0 mmol/l), hypertriglyceridemia (≥1.7 mmol/l), reduced level of high-density lipoprotein cholesterol (≤1.0 mmol/l for men and ≤1.2 mmol/l for women) [18]. Patients with either fasting plasma glucose (FPG) ≥ 6.1-6.9 mmol/L (impaired FPG) or 2-hour plasma glucose (2hPG) ≥ 7.8-11.0 mmol/L (impaired glucose tolerance) or HBA1c 6.0-6.4% were diagnosed with prediabetes and subjects with FPG ≥ 7.0 and/or 2hPG ≥ 11.1 were considered diabetics. Patients with HBA1c ≥ 6.5% underwent testing to confirm diabetes [19]. These patients were grouped as impaired carbohydrate metabolism. Patients were considered to have a diagnosis of GERD if their GerdQ questionnaire score was ≥ 8. Written permission from AstraZeneca was obtained to use GerdQ [20]. Liver steatosis was diagnosed based on ultrasound scan results in patients with increased parenchymal echogenicity and disturbed visibility of vascular structures in the liver compared with those in the kidney [21]. These patients were diagnosed with Non-alcoholic fatty liver disease (NAFLD). The clinical picture and ultrasound findings were considered to determine the presence of chronic pancreatitis and cholecystitis in the patients. The diagnosis of peptic ulcer disease and functional disorders of the gastrointestinal system was made if there was evidence in the medical records based on the patient interview and the presence of relevant symptoms and signs. Based on the STOP-BANG tool, obstructive sleep apnea syndrome (OSA) was considered in individuals who met the high-risk score [22]. Anemia was diagnosed based on hemoglobin levels [23].

The presence of dyslipidemia or impaired carbohydrate metabolism was recorded as a Metabolic Disorder (MD). The remaining diseases were documented based on the data provided by the patients.

Ethical approval

The study was approved by the Local Ethics Committee of Kazan State Medical University (Protocol No. 6, dated June 22, 2021).

Statistical analysis

Statistical analyses were performed using IBM SPSS® Statistics version 26 (IBM Corp., Armonk, NY, USA). The normality of continuous variables was tested using the Kolmogorov-Smirnov test. As the data were not normally distributed, non-parametric analytical methods were used. Continuous variables are presented as medians and interquartile ranges [IQR, 25th-75th percentile]. Descriptive statistics were used to obtain frequencies and percentages for categorical variables. The Mann‐Whitney U‐test was used to compare two independent groups, and the Kruskal‐Wallis test to compare three or more groups. Binary logistic regression was performed to quantify the contribution of BMI, abdominal obesity, and visceral fat to the presence of therapeutic comorbidities. Differences between the groups were considered statistically significant at p < 0.05.

## Results

**Figure 1:**
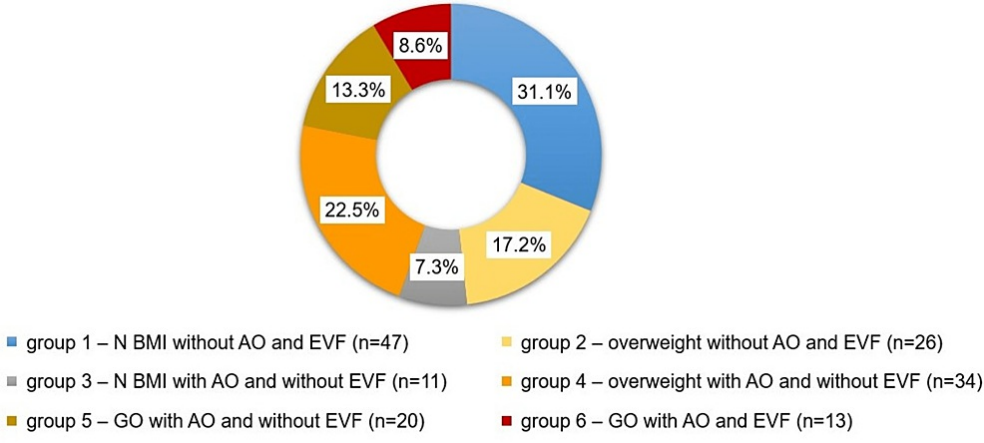
Participant distribution across different obesity phenotypes. N: normal; BMI: body mass index; AO: abdominal obesity; EVF: excess visceral fat level; GO: general obesity.

Elevated BMI was observed in 61.6% of the cases, including overweight in 39.7% (n=60) and obese in 21.9% (n=33). AO was detected in every second patient (n=78; 51.6%). Increased WC was observed in 49.0% of cases (n=74) and WHR in 32.5% of cases (n=49). Four patients had normal WC values but an elevated WHR. The frequency of EVF was lower (n=13; 8.6%), and the median visceral fat level was 6 [IQR: 4-9]. Furthermore, we divided the participants according to different obesity phenotypes, the results of which are presented in Figure 1.

The five most frequently observed conditions in the general cohort were dyslipidemia (71.5%; n=108), disorders of the gastrointestinal tract (53.0%; n=80), cardiovascular disease (46.4%; n=70), musculoskeletal diseases (40.4%; n=61), and impaired carbohydrate metabolism (25.2%; n=38). The detailed characteristics of the diseases within their subgroups are presented in Table 1. Due to the low frequency and/or sparse distribution across the groups, the following conditions were excluded from the table: COPD (1 patient), asthma (1 patient), type 2 diabetes mellitus (2 patients), and obstructive sleep apnea (17 patients).

**Table 1:** Prevalence of disease in individuals with different phenotypes of obesity (n=151). N: normal; BMI: body mass index; AO: abdominal obesity; EVF: excess level of visceral fat; GO: general obesity; HTN: hypertension; BP: blood pressure; COPD: Chronic obstructive pulmonary disease; T.ch: total cholesterol; LDL-C – low-density lipoprotein cholesterol; HDL-C – high-density lipoprotein cholesterol; GIT: gastrointestinal tract; GERD: Gastroesophageal reflux disease; NAFLD: Non-alcoholic fatty liver disease; ↑ increased, ↓ reduced; *Allergic rhinitis, allergic contact eczema, neurodermatitis, food allergy or urticaria; NA: Not available/applicable.

Disease	Total	N BMI without AO, EVF	Overweight without AO, EVF	N BMI + АО without EVF	Overweight + АО without EVF	GO + АО without EVF	GO + АО + EVF	р_1-2_	р_1-3_	р_1-4_	р_1-5_	р_1-6_
n (%)		n=47 (31.1)	n=26 (17.2)	n=11 (7.3)	n=34 (22.5)	n=20 (13.2)	n=13 (8.6)					
Group number		1	2	3	4	5	6					
Male / Female	64 (42.4) / 87(57.6)	19 (40.4) /28 (59.6)	16 (61.5)/10 (38.5)	1 (9.1) / 10 (90.9)	16 (47.1) / 18 (52.9)	7 (35.0) / 13 (65.0)	5 (38.5) / 8 (61.5)	0.244	0.244	0.745	0.781	0.244
Any cardiovascular disease	70 (46.4)	12 (25.5)	5 (19.2)	3 (27.3)	25 (73.5)	14 (70.0)	11 (84.6)	0.677	0.906	<0.001	0.002	<0.001
HTN	61 (40.4)	7 (14.9)	5 (19.2)	2 (18.2)	22 (64.7)	14 (70.0)	11 (84.6)	0.790	0.842	<0.001	<0.001	<0.001
Suspected cases of HTN	16 (10.6)	1 (3.3)	2 (14.3)	0	8 (88.9)	3 (50.0)	2 (66.7)	0.224	NA	<0.001	0.002	0.001
Uncontrolled HTN	41 (27.2)	5 (10.6)	2 (7.7)	2 (18.2)	15 (44.1)	10 (50.0)	7 (53.8)	0.731	0.667	0.003	0.003	0.003
High normal BP	18 (11.9)	5 (10.6)	5 (19.2)	0	5 (14.7)	2 (10.0)	1 (7.7)	0.070	NA	0.820	0.711	0.925
Varicose veins of the lower extremities	12 (7.9)	4 (8.5)	0	1 (9.1)	4 (11.8)	2 (10.0)	1 (7.7)	NA	0.951	0.951	0.951	0.951
Anemia	12 (7.9)	2 (4.3)	2 (7.7)	3 (27.3)	3 (8.8)	1 (5.0)	1 (7.7)	0.965	0.215	0.965	0.965	0.965
Any dyslipidemia	108 (71.5)	27 (57.4)	14 (53.8)	10 (90.9)	27 (79.4)	20 (100.0)	10 (76.9)	0.852	0.096	0.096	NA	0.335
↑ T.ch	71 (50.7)	16 (35.6)	9 (36.0)	7 (63.6)	20 (64.5)	14 (70.0)	5 (62.5)	0.970	0.009	0.013	0.010	0.151
↑LDL-C	82 (61.7)	7 (14.9)	4 (15.4)	3 (27.3)	8 (23.5)	7 (35.0)	5 (38.5)	0.919	0.311	0.008	0.002	0.002
↓HDL-C	34 (22.5)	20 (46.5)	11 (47.8)	7 (63.6)	24 (77.4)	16 (88.9)	4 (57.1)	0.955	0.328	0.323	0.064	0.060
Hypertriglyceridemia	23 (15.2)	1 (2.1)	1 (3.8)	0	8 (23.5)	6 (30.0)	7 (53.8)	0.667	0.002	NA	0.001	<0.001
Any impaired carbohydrate metabolism	38 (25.2)	5 (10.6)	5 (19.2)	2 (18.2)	13 (38.2)	6 (30.0)	7 (53.8)	0.307	0.489	0.003	0.005	0.001
Prediabetes	36 (23.8)	5 (10.6)	5 (19.2)	2 (18.2)	13 (38.2)	5 (25.0)	6 (46.2)	0.291	0.429	0.001	0.032	<0.001
Any GIT disease	80 (53.0)	26 (55.3)	12 (46.2)	4 (36.4)	17 (50.0)	11 (55.0)	10 (76.9)	0.755	0.644	0.795	0.981	0.598
Any functional disorders of the upper GIT	33 (21.9)	15 (31.9)	5 (19.2)	3 (27.3)	4 (11.8)	6 (30.0)	0	0.611	0.877	0.347	0.877	NA
GERD	18 (12)	1 (2.1)	1 (3.8)	1 (9.1)	6 (17.6)	5 (25.0)	4 (30.8)	0.667	0.255	0.014	0.003	0.001
Peptic ulcer disease	15 (9.9)	5 (10.6)	2 (7.7)	0	6 (17.6)	0	2 (15.4)	0.819	NA	0.819	NA	0.819
Chronic pancreatitis	15 (9.9)	3 (6.4)	2 (7.7)	1 (9.1)	2 (5.9)	2 (10.0)	5 (38.5)	0.832	0.750	0.926	0.606	0.003
Gall bladder disease	14 (9.3)	3 (6.4)	2 (7.7)	0	2 (5.9)	3 (15.0)	4 (30.8)	0.926	0.924	NA	0.465	0.111
Constipation syndrome	10 (6.6)	4 (8.5)	1 (3.8)	1 (9.1)	3 (8.8)	1 (5.0)	0	0.978	0.978	0.978	0.978	NA
Any functional diarrhea	6 (4.0)	1 (2.1)	2 (7.7)	0	1 (2.9)	0	2 (15.4)	0.503	NA	0.816	NA	0.313
Any diseases of the urinary system	6 (4.0)	2 (4.3)	1 (3.8)	1 (9.1)	2 (5.9)	0	0	0.933	0.886	0.886	NA	NA
Urolithiasis	3 (2.0)	1(2.1)	0	1 (9.1)	1 (2.9)	0	0	NA	0.367	0.667	NA	NA
Pyelonephritis	3 (2.0)	1 (2.1)	1 (3.8)	0	1 (2.9)	0	0	0.237	NA	0.377	NA	NA
Any musculoskeletal disease	61 (40.4)	16 (34.0)	12 (46.2)	6 (54.5)	13 (38.2)	8 (40.0)	6 (46.2)	0.839	0.839	0.839	0.839	0.839
Osteoarthritis	38 (25.2)	7 (14.9)	7 (26.9)	2 (18.2)	11 (32.4)	6 (30.0)	5 (38.5)	0.792	0.857	0.466	0.762	0.466
Degenerative-dystrophic diseases of the spine	41 (27.2)	12 (25.5)	8 (30.8)	6 (54.5)	7 (20.6)	5 (25.0)	3 (23.1)	0.963	0.422	0.963	0.963	0.963
Atopic disease*	36 (23.8)	13 (27.7)	7 (26.9)	2 (18.2)	6 (17.6)	6 (30.0)	2 (15.4)	0.968	0.952	0.968	0.968	0.968

**Figure 2:**
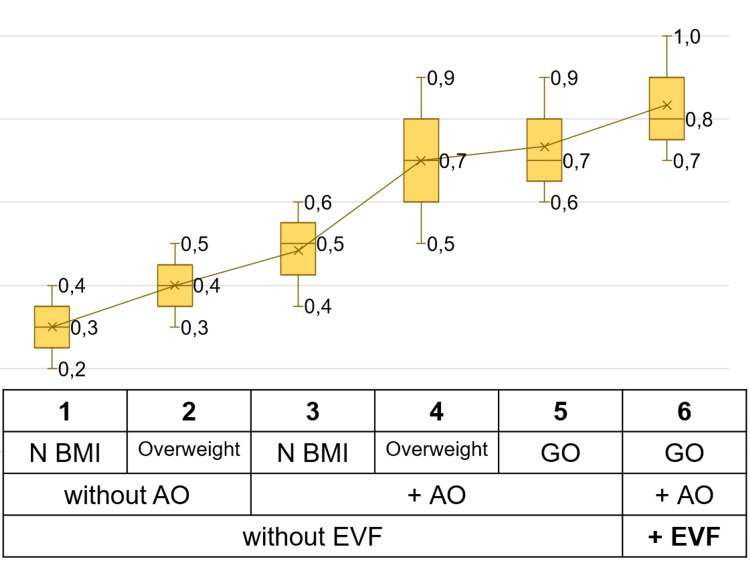
Comorbidity among groups with different phenotypes of obesity. N: normal; BMI: body mass index; AO: abdominal obesity; EVF: excess visceral fat level; GO: general obesity.

Next, we examined comorbidities in the groups with different obesity phenotypes (Table 1). The simple addition of the disease calculated comorbidity. Data are presented as the median and interquartile range [25-75%]. The median number of pathological combinations in the general cohort was five [IQR: 3-7]. The highest number of pathologies detected for each patient was 13. As the group number increased, the median number of comorbidities also increased (Figure 2). The median comorbidity in the sixth group with GO, AO, and EVF was 8 [IQR: 7-10], which was significantly higher than that in groups with normal BMI or overweight but without AO and EVF (group one - 3 [IQR: 2-4], group two - 4 [IQR: 3-5]; p<0.001 and p<0.001, respectively), and normal weight and AO without EVF (group three - 5 [IQR: 3,5-6]; p=0.009). The median comorbidity of patients with overweight (group four - 7 [IQR: 5-9]) or GO (group five - 7 [IQR: 9-6]) and AO and without EVF was higher than those in groups one, two, and three (p<0.001, p<0.001, p=0.051 and p<0.001, p<0.001, p=0.021, respectively) (Figure 2).

Any cardiovascular disease was present in more than half of the participants in the groups with AO and elevated BMI (group four - 73.5%, group five - 70.0%, and group six - 84.6%). This was statistically significant compared with the groups with normal BMI or overweight without AO and EVF (group one - 25.5%, group two - 19.2%, group three - 27.3%; p<0.001). The increase in the frequency of HTN with increasing group number was statistically significant (group one - 14.9%, group two - 19.2%, group three - 18.2%, group four - 64.7%, group five - 70.0%, group six - 84.6%; p<0.001). In every second patient with GO, target BP levels were not achieved (group five - 50.0%, group six - 53.8%); this was statistically significant compared with group one (10.6%, p=0.003 and p=0.003, respectively) and group two (7.7%, p=0.004 and p=0.004, respectively).

The frequency of any dyslipidemia was high in all participant groups. Regardless of BMI, participants in the AO group had increased total cholesterol levels in over 50% of cases. Elevated total cholesterol levels were found in 63.6%, 64.5%, and 70% of groups three, four, and five, respectively, which were significantly higher than those in group one (n=16; 35.6%, p=0.090, p=0.013, and p=0.010, respectively).

Digestive tract diseases were noted in over half of the subjects with general obesity, AO, and EVF (group six - 76.9%) (Table 1). GERD occurred in every fourth patient with general obesity and AO (group five - 25.0%) and every third individual of the group with EVF (group six - 30.8%), which was more frequent compared to individuals from the group with normal BMI (first group - 2.1%; p=0.003 and p=0.001, respectively) and overweight without AO (second group - 3.8% p=0.035 and p=0.018, respectively). Chronic pancreatitis was found in 38.5% of patients with EVF in the sixth group, which was significantly higher than that in the overweight and AO (fourth group - 5.9%; p=0.005) group, as well as in those without AO with normal BMI (first group - 6.4%; p=0.003) and overweight (second group - 7.7%; p=0.018). NAFLD was diagnosed in every fifth patient with GO, AO, and EVF (sixth group n=3; 23.1%).

In the final step, we conducted multiple regression analysis using a stepwise approach to determine the independent predictors for each comorbidity; the results are presented in Table 2.

**Table 2:** Predictors associated with obesity phenotypes in different comorbidity obtained using multiple logistic regression analysis (n=151). BMI: body mass index; AO: abdominal obesity; HTN: hypertension; T.ch: total cholesterol; LDL-C – low-density lipoprotein cholesterol; HDL-C – high-density lipoprotein cholesterol; Tg – triglycerides; NAFLD: Non-alcoholic fatty liver disease; GERD: Gastroesophageal reflux disease; OSA: obstructive sleep apnea syndrome; ↑ increased; ↓ reduced; – the predictor was not included in the model; the preliminary final models was correctly specified (p < 0.05).

Сomorbidity	Predictor Variables	OR	95% CI	р	R^2 ^(Nagelkerke)	χ^2^
HTN	BMI (kg/m^2^)	1.18	1.10-1.30	0.002	37.0%	47.2
	АО (0 – no, 1 – yes)	3.65	1.50-9.10	0.006		
↑ T.ch	age, years	2.32	1.07-5.02	<0.001	32.1%	39.8
	АО (0 – no, 1 – yes)	1.11	1.06-1.16	0.033		
↑ HDL-C	age, years	1.10	1.04-1.16	<0.001	31.1%	34.6
	level of visceral fat	1.14	0.99-1.31	0.075		
Hypertriglyceridemia	АО (0 – no, 1 – yes)	6.04	1.18-30.8	0.031	27.0%	28.7
	level of visceral fat	1.19	1.03-1.37	0.020		
↓LDL-C	age, years	0.91	0.86-0.96	<0.001	21.4%	22.2
	BMI (kg/m^2^)	1.08	0.99-1.19	0.077		
	АО (0 – no, 1 – yes)	2.91	0.95-8.93	0.063		
NAFLD	age, years	1.13	0.99-1.28	0.073	32.2%	17.5
	level of visceral fat	1.35	1.09-1.68	0.007		
Chronic pancreatitis	level of visceral fat	1.26	1.09-1.45	0.001	14.9%	11.1
GERD	АО (0 – no, 1 – yes)	9.16	2.03-41.4	0.004	15.7%	12.0
Prediabetes	level of visceral fat	1.18	1.07-1.30	0.001	10.7%	15.9
OSA	gender (0 -female, 1- male)	3.49	1.04-11.7	0.043	33.4%	32.8
	level of visceral fat	1.41	1.19-1.66	<0.001		

**Figure 3:**
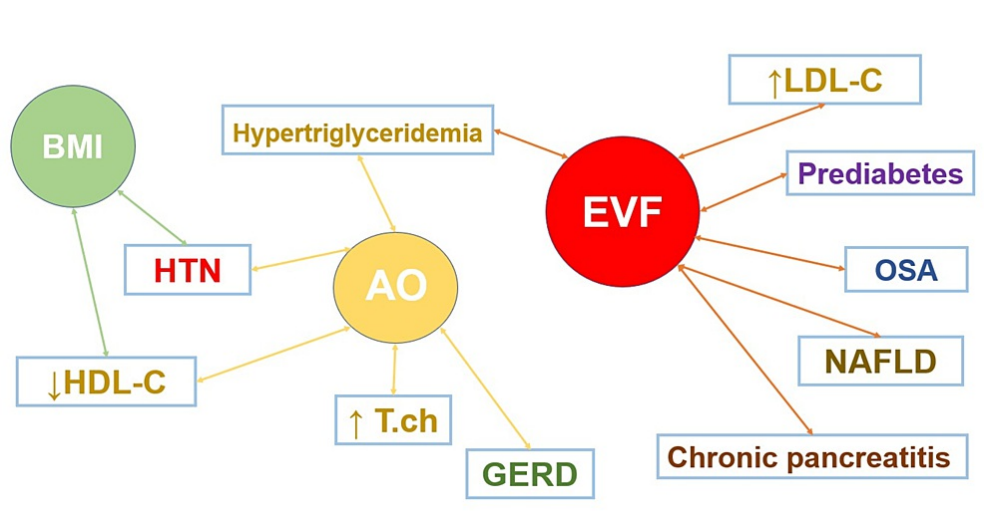
Participant distribution across different obesity phenotypes. N: normal; BMI: body mass index; AO: abdominal obesity; EVF: excess level of visceral fat; GO: general obesity; T.ch: total cholesterol; LDL-C – low-density lipoprotein cholesterol; HDL-C – high-density lipoprotein cholesterol; NAFLD: Non-alcoholic fatty liver disease; GERD: Gastroesophageal reflux disease; OSA: obstructive sleep apnea syndrome; ↑ increased; ↓ reduced.

While BMI was significantly associated only with arterial hypertension, the level of visceral fat was associated with most comorbidities (OSA, NAFLD, chronic pancreatitis, hypertriglyceridemia, and prediabetes), followed by abdominal obesity (GERD, hypertriglyceridemia, arterial hypertension, hypercholesterolemia). A schematic representation of these associations is presented in Figure 3.

## Discussion

Our data demonstrate a high incidence of metabolic disorders, arterial hypertension, and gastrointestinal tract and musculoskeletal system diseases in young and middle-aged patients, who are primary contributors to the workforce. Our results are consistent with current ideas regarding obesity as a heterogeneous disease, which is associated with the development of several comorbid pathologies [24,25].

The results showed that AO was associated with the risk of developing arterial hypertension and GERD. This could be explained by the fact that abdominal obesity without general obesity is associated with a greater fat mass than only general obesity without abdominal obesity because BMI is calculated, including muscle mass. The level of visceral fat, as measured by bioimpedance analysis, was associated with the development of NAFLD, chronic pancreatitis, obstructive sleep apnea syndrome, hypertriglyceridemia, lowering HDL cholesterol, and рrediabetes. This aspect is even more relevant because the development of comorbidity is influenced not only by the volume but also by the quality of subcutaneous and visceral fat [4,5,26]. In addition, the absence of a significant effect of BMI on the development of certain pathological conditions is consistent with the finding that individuals with normal BMI and AO may have a higher percentage of visceral fat [3].

Our results present data obtained using simple anthropometric methods for identifying obesity types. Our findings could improve the effectiveness of performing comprehensive patient evaluation and designing management strategies from a primary prevention perspective.

The study's strength lies in the detailed clinical examination, analysis of medical records, and laboratory and instrumental tests for a good sample size that brings credibility to the results. Our study has some limitations. First, the case-control design of the study could theoretically result in a selection bias. Second, during categorization, the participants were grouped into six phenogroups, resulting in some groups with a smaller sample. Lastly, this was a one-time study, thus limiting prospective evaluations.

## Conclusions

In working-age people, group 1 and 4 phenotypes with normal BMI, without AO and EVF, and overweight with AO and without EVF were more frequent than other phenotypes. Phenotype 6, i.e., general obesity with abdominal obesity and excess level of visceral fat, was identified in almost every tenth patient. The median of concomitant diseases increased as the combination of various obesities (general, abdominal, and visceral fat levels) increased. We have also established the contribution of various obesity phenotypes in forming certain types of comorbidities. Abdominal obesity and visceral fat were associated with the most comorbid conditions. However, the individual types of these comorbidities were not the same. While abdominal obesity is a predictor for increased levels of total cholesterol, hypertriglyceridemia, reduced HDL-C levels, and GERD, the level of visceral fat was the main contributor to the presence of prediabetes, NAFLD, chronic pancreatitis and OSA, hypertriglyceridemia and elevated LDL-C levels. Thus, a comprehensive examination of patients to identify the obesity phenotype, including the determination of WC and the level of visceral fat, can help develop better measures for primary and secondary prevention of comorbidities.
